# *N*′-Benzoyl-5-methyl-1,3-diphenyl-1*H*-pyrazole-4-carbohydrazide

**DOI:** 10.1107/S1600536813029528

**Published:** 2013-11-13

**Authors:** Chandra, N. Srikantamurthy, Shamantha Kumar, B. H. Doreswamy, K. B. Umesha, M. Mahendra

**Affiliations:** a Department of Studies in Physics, Manasagangotri, University of Mysore, Mysore 570 006, India; b Department of Chemistry, Yuvaraja’s College, University of Mysore, Mysore 570 005, India; c Department of Physics, SJB Institute of Technology, Kengeri, Bangalore 560 060, India

## Abstract

In the title compound, C_24_H_20_N_4_O_2_, the pyrazole ring makes dihedral angles of 47.57 (10)° and 30.56 (11)° with its N-bound and C-bound phenyl groups, respectively. The C—N—N—C group that links the two carbonyls has a torsion angle of 81.5 (2)°. The torsion angles between the carbonyl groups and their adjacent pyrazole and phenyl rings are 125.89 (19) and 164.22 (17)°, respectively. In the crystal, pairs of mol­ecules are linked by N—H⋯O hydrogen bonds into *R*_2_^2^(10) ring motifs, which in turn link to form chains that propagate parallel to the *c-*axis direction.

## Related literature

For the biological activity of pyrazoles, see: Cunico *et al.* (2006 ▶); Farag *et al.* (2008 ▶); Sharma *et al.* (2010 ▶); Patel *et al.* (2004 ▶). For the synthesis of pyrazoles, see: Shridevi Doddaramappa *et al.* (2013 ▶). For bond-length and angle data in a related structure, see: Chandra *et al.* (2012 ▶).
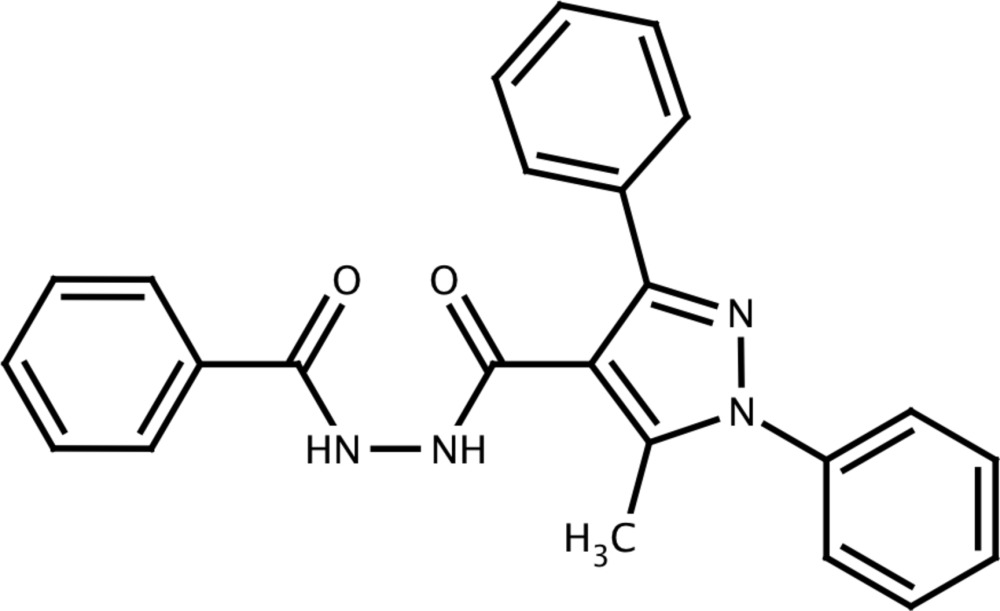


## Experimental

### 

#### Crystal data

C_24_H_20_N_4_O_2_
*M* *_r_* = 396.44
Monoclinic, 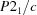

*a* = 22.399 (15) Å
*b* = 11.180 (8) Å
*c* = 8.190 (6) Å
β = 97.378 (12)°
*V* = 2034 (2) Å^3^
*Z* = 4
Mo *K*α radiation
μ = 0.09 mm^−1^
*T* = 293 K
0.30 × 0.25 × 0.20 mm

#### Data collection

Bruker APEXII CCD area-detector diffractometer
18837 measured reflections
3601 independent reflections
2793 reflections with *I* > 2σ(*I*)
*R*_int_ = 0.039

#### Refinement

*R*[*F* ^2^ > 2σ(*F* ^2^)] = 0.040
*wR*(*F* ^2^) = 0.104
*S* = 1.04
3601 reflections
280 parameters
H atoms treated by a mixture of independent and constrained refinement
Δρ_max_ = 0.16 e Å^−3^
Δρ_min_ = −0.16 e Å^−3^

### 

Data collection: *APEX2* (Bruker, 2009 ▶); cell refinement: *SAINT* (Bruker, 2009 ▶); data reduction: *SAINT*; program(s) used to solve structure: *SHELXS97* (Sheldrick, 2008 ▶); program(s) used to refine structure: *SHELXL97* (Sheldrick, 2008 ▶); molecular graphics: *PLATON* (Spek, 2009 ▶); software used to prepare material for publication: *SHELXL97*.

## Supplementary Material

Crystal structure: contains datablock(s) global, I. DOI: 10.1107/S1600536813029528/pk2500sup1.cif

Structure factors: contains datablock(s) I. DOI: 10.1107/S1600536813029528/pk2500Isup2.hkl

Supplementary material file. DOI: 10.1107/S1600536813029528/pk2500Isup3.cml

Additional supplementary materials:  crystallographic information; 3D view; checkCIF report

## Figures and Tables

**Table 1:** Hydrogen-bond geometry (Å, °)

*D*—H⋯*A*	*D*—H	H⋯*A*	*D*⋯*A*	*D*—H⋯*A*
N21—H21⋯O20^i^	0.88 (2)	2.05 (2)	2.925 (3)	174.5 (17)
N22—H22⋯O24^ii^	0.90 (2)	1.98 (2)	2.864 (3)	167.7 (17)

Symmetry codes: (i) 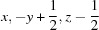; (ii) 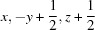.

**Fig. 1.**
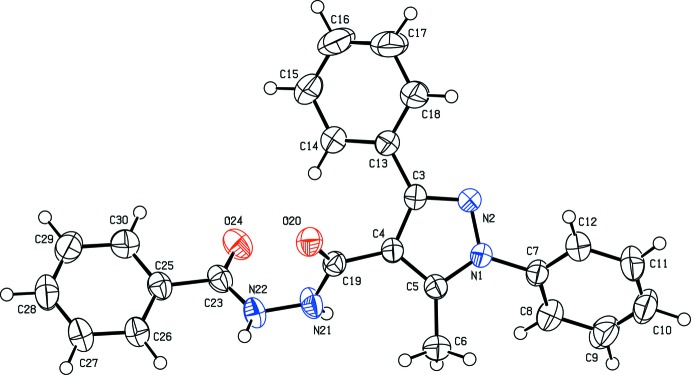
Perspective diagram of the molecule with 50% probability displacement ellipsoids.

**Fig. 2.**
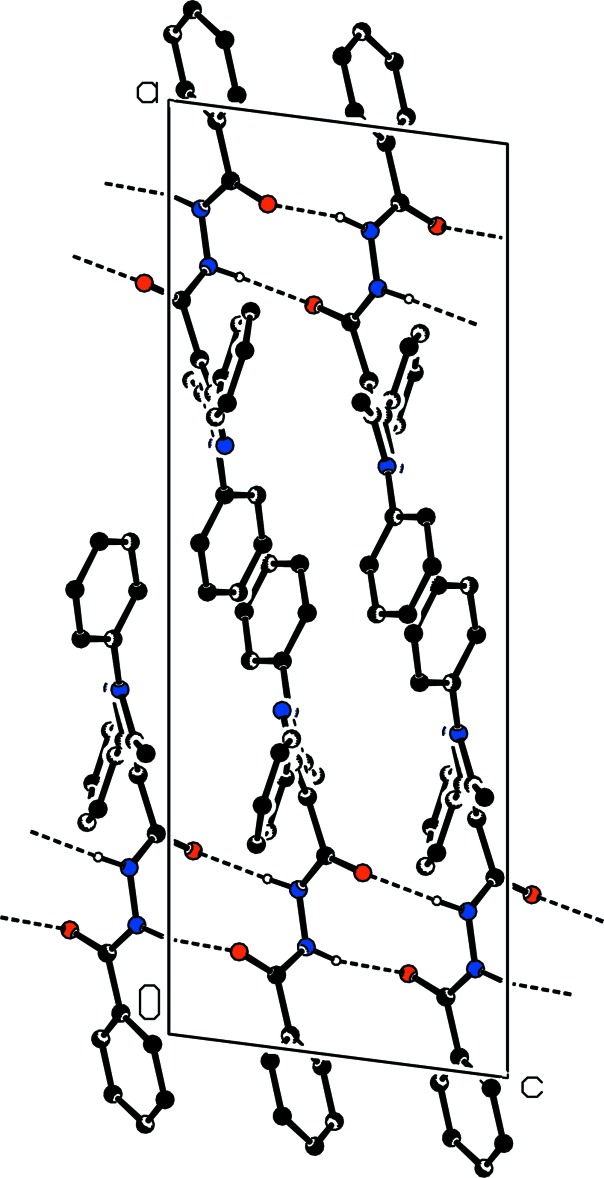
Packing diagram of the structure viewed down the 'b' axis.

## supplementary crystallographic information

### 1. Comment

Compounds that contain the pyrazole moiety are known to exhibit a wide range of biological properties such as anti-malarial (Cunico *et al.*, 2006), anti-tumor (Farag *et al.*, 2008) and anti-inflammatory activities (Sharma *et al.*, 2010). In addition, pyrazoles have a wide variety of application in the agrochemical and pharmaceutical industries (Patel *et al.*, 2004). Recently we have reported the synthesis of substituted pyrazoles (Shridevi Doddaramappa *et al.*, 2013). As an extension of our work on the structural characterization of pyrazoles, the title compound was prepared and characterized by single-crystal X-ray diffraction.

In the molecular structure of the title compound (Fig. 1), the bond lengths and angles are generally within normal ranges and are comparable to those in a related structure (Chandra *et al.*, 2012). The pyrazole moiety makes a torsion angle of 47.57 (10)° and 30.56 (11)° with the N-bound phenyl (C8–C13) and C-bound phenyl (C14–C19) groups, respectively. Also, the pyrazole ring makes a torsion angle of 51.36 (10)° with the amide group (C4/C19/O20/N21). The C-N-N-C group that links the two carbonyls has a torsion angle of 81.5 (2)°. Torsion angles between the carbonyl groups and their adjacent pyrazole and phenyl rings are 125.89 (19)° and 164.22 (17)°, respectively. In the crystal, pairs of molecules are linked by N—H···O hydrogen bonds into *R*_2_^2^(10) ring motifs, which in turn link to form chains that propagate parallel to the *c* axis (Fig. 2).

### 2. Experimental

To pyrazole 4-carboxylic acid in CH_2_Cl_2_ (5 ml), ethyl-(*N*',*N*'-dimethylamino)propylcarbodiimide hydrochloride (EDC·HCl, 1.2 mmol) and 1-hydroxybenzotriazole (HOBt, 1.2 mmol) and then benzohydrazide (0.136 g, 1.0 mmol) was added and stirred at 25° C for 8–12 h. After completion of the reaction, the reaction mixture was extracted with ethyl acetate and the combined organic phase was washed with brine and dried over anhydrous sodium sulfate. Ethyl acetate was distilled off and the residue thus obtained was purified by column chromatography to give a white solid. The typical size of the block-shaped crystals was 0.30 × 0.25 × 0.20 mm.

### 3. Refinement

Carbon-bound H atoms were positioned geometrically and allowed to ride on their parent atoms with C–H distances in the range of 0.93 to 0.96 Å, respectively. *U*_iso_(H) values were set to 1.2–1.5*U*_eq_ of the attached atom. The coordinates of H-atoms attached to nitrogen were allowed to refine.

### Crystal data

**Table:**

C_24_H_20_N_4_O_2_	*F*(000) = 832
*M**_r_* = 396.44	*D*_x_ = 1.295 Mg m^−^^3^
Monoclinic, *P*2_1_/*c*	Mo *K*α radiation, λ = 0.71073 Å
Hall symbol: -P 2ybc	Cell parameters from 3601 reflections
*a* = 22.399 (15) Å	θ = 1.8–25.1°
*b* = 11.180 (8) Å	µ = 0.09 mm^−^^1^
*c* = 8.190 (6) Å	*T* = 293 K
β = 97.378 (12)°	Block, white
*V* = 2034 (2) Å^3^	0.30 × 0.25 × 0.20 mm
*Z* = 4	

### Data collection

**Table:**

Bruker APEXII CCD area-detector diffractometer	*R*_int_ = 0.039
ω and φ scans	θ_max_ = 25.1°, θ_min_ = 1.8°
18837 measured reflections	*h* = −26→26
3601 independent reflections	*k* = −13→13
2793 reflections with *I* > 2σ(*I*)	*l* = −9→9

### Refinement

**Table:**

Refinement on *F*^2^	Primary atom site location: structure-invariant direct methods
Least-squares matrix: full	Secondary atom site location: difference Fourier map
*R*[*F*^2^ > 2σ(*F*^2^)] = 0.040	Hydrogen site location: inferred from neighbouring sites
*wR*(*F*^2^) = 0.104	H atoms treated by a mixture of independent and constrained refinement
*S* = 1.04	*w* = 1/[σ^2^(*F*_o_^2^) + (0.0447*P*)^2^ + 0.4179*P*] where *P* = (*F*_o_^2^ + 2*F*_c_^2^)/3
3601 reflections	(Δ/σ)_max_ < 0.001
280 parameters	Δρ_max_ = 0.16 e Å^−^^3^
0 restraints	Δρ_min_ = −0.16 e Å^−^^3^

### Special details

**Table:**

Geometry. Bond distances, angles *etc*. have been calculated using the rounded fractional coordinates. All su's are estimated from the variances of the (full) variance-covariance matrix. The cell e.s.d.'s are taken into account in the estimation of distances, angles and torsion angles
Refinement. Refinement on *F*^2^ for ALL reflections except those flagged by the user for potential systematic errors. Weighted *R*-factors *wR* and all goodnesses of fit *S* are based on *F*^2^, conventional *R*-factors *R* are based on *F*, with *F* set to zero for negative *F*^2^. The observed criterion of *F*^2^ > σ(*F*^2^) is used only for calculating -*R*-factor-obs *etc*. and is not relevant to the choice of reflections for refinement. *R*-factors based on *F*^2^ are statistically about twice as large as those based on *F*, and *R*-factors based on ALL data will be even larger.

### Fractional atomic coordinates and isotropic or equivalent isotropic displacement parameters (Å^2^)

**Table:**

	*x*	*y*	*z*	*U*_iso_*/*U*_eq_	
O20	0.19942 (5)	0.37324 (10)	0.57237 (14)	0.0459 (4)	
O24	0.09764 (5)	0.37966 (12)	0.20756 (15)	0.0542 (5)	
N1	0.36194 (5)	0.24500 (11)	0.35566 (17)	0.0386 (4)	
N2	0.36205 (6)	0.36600 (12)	0.33447 (17)	0.0412 (5)	
N21	0.17262 (6)	0.23024 (14)	0.3828 (2)	0.0464 (5)	
N22	0.11216 (6)	0.23944 (14)	0.4056 (2)	0.0453 (5)	
C3	0.30808 (7)	0.40197 (14)	0.3675 (2)	0.0372 (5)	
C4	0.27321 (7)	0.30316 (14)	0.40903 (19)	0.0354 (5)	
C5	0.30905 (7)	0.20387 (14)	0.3993 (2)	0.0365 (5)	
C6	0.29699 (8)	0.07602 (15)	0.4324 (2)	0.0495 (6)	
C7	0.41505 (7)	0.17858 (14)	0.3349 (2)	0.0408 (5)	
C8	0.41110 (8)	0.07649 (17)	0.2407 (2)	0.0532 (7)	
C9	0.46268 (10)	0.01246 (19)	0.2225 (3)	0.0669 (8)	
C10	0.51756 (10)	0.0515 (2)	0.2948 (3)	0.0713 (9)	
C11	0.52135 (9)	0.1549 (2)	0.3862 (3)	0.0696 (8)	
C12	0.47012 (8)	0.21913 (17)	0.4085 (3)	0.0555 (7)	
C13	0.29037 (7)	0.52833 (14)	0.3435 (2)	0.0408 (5)	
C14	0.23106 (8)	0.55850 (16)	0.2907 (2)	0.0485 (6)	
C15	0.21515 (9)	0.67597 (18)	0.2585 (3)	0.0660 (8)	
C16	0.25768 (11)	0.76367 (19)	0.2758 (4)	0.0855 (10)	
C17	0.31656 (11)	0.73563 (19)	0.3275 (4)	0.0847 (9)	
C18	0.33299 (9)	0.61860 (17)	0.3625 (3)	0.0612 (7)	
C19	0.21258 (7)	0.30730 (14)	0.4629 (2)	0.0360 (5)	
C23	0.07792 (7)	0.32262 (15)	0.3178 (2)	0.0410 (6)	
C25	0.01545 (7)	0.34010 (15)	0.3583 (2)	0.0402 (5)	
C26	−0.01345 (7)	0.26058 (17)	0.4520 (2)	0.0460 (6)	
C27	−0.07082 (8)	0.28462 (19)	0.4886 (2)	0.0558 (7)	
C28	−0.09995 (8)	0.38720 (19)	0.4304 (3)	0.0584 (7)	
C29	−0.07218 (9)	0.46552 (18)	0.3351 (3)	0.0593 (7)	
C30	−0.01473 (8)	0.44250 (17)	0.2992 (2)	0.0523 (7)	
H6A	0.28550	0.03520	0.33010	0.0740*	
H6B	0.26500	0.07050	0.49970	0.0740*	
H6C	0.33270	0.03980	0.48870	0.0740*	
H8	0.37390	0.05060	0.18930	0.0640*	
H9	0.46000	−0.05760	0.16080	0.0800*	
H10	0.55220	0.00830	0.28230	0.0860*	
H11	0.55880	0.18220	0.43370	0.0840*	
H12	0.47290	0.28840	0.47190	0.0670*	
H14	0.20180	0.49910	0.27700	0.0580*	
H15	0.17510	0.69550	0.22460	0.0790*	
H16	0.24670	0.84270	0.25250	0.1020*	
H17	0.34560	0.79560	0.33910	0.1020*	
H18	0.37300	0.60020	0.39920	0.0730*	
H21	0.1796 (8)	0.1945 (17)	0.291 (3)	0.058 (6)*	
H22	0.1019 (8)	0.2004 (17)	0.494 (3)	0.059 (6)*	
H26	0.00590	0.19060	0.49040	0.0550*	
H27	−0.08970	0.23140	0.55260	0.0670*	
H28	−0.13840	0.40360	0.45550	0.0700*	
H29	−0.09220	0.53440	0.29460	0.0710*	
H30	0.00380	0.49610	0.23500	0.0630*	

### Atomic displacement parameters (Å^2^)

**Table:**

	*U*^11^	*U*^22^	*U*^33^	*U*^12^	*U*^13^	*U*^23^
O20	0.0476 (7)	0.0487 (7)	0.0441 (7)	0.0038 (6)	0.0161 (6)	−0.0043 (6)
O24	0.0513 (8)	0.0636 (8)	0.0506 (8)	−0.0096 (6)	0.0173 (6)	0.0017 (6)
N1	0.0320 (7)	0.0342 (7)	0.0510 (9)	0.0040 (6)	0.0106 (6)	0.0026 (6)
N2	0.0347 (8)	0.0331 (8)	0.0569 (9)	0.0019 (6)	0.0101 (6)	0.0039 (6)
N21	0.0315 (8)	0.0596 (10)	0.0505 (10)	−0.0040 (7)	0.0146 (7)	−0.0125 (8)
N22	0.0311 (8)	0.0593 (10)	0.0481 (9)	−0.0011 (7)	0.0148 (7)	0.0007 (8)
C3	0.0316 (9)	0.0384 (9)	0.0419 (9)	0.0026 (7)	0.0058 (7)	−0.0004 (7)
C4	0.0307 (8)	0.0373 (9)	0.0386 (9)	0.0015 (7)	0.0063 (7)	0.0010 (7)
C5	0.0329 (8)	0.0382 (9)	0.0390 (9)	−0.0005 (7)	0.0065 (7)	0.0015 (7)
C6	0.0493 (11)	0.0393 (10)	0.0612 (12)	0.0005 (8)	0.0121 (9)	0.0081 (8)
C7	0.0349 (9)	0.0406 (9)	0.0486 (10)	0.0074 (7)	0.0123 (8)	0.0073 (8)
C8	0.0497 (11)	0.0545 (11)	0.0563 (12)	0.0112 (9)	0.0100 (9)	−0.0039 (9)
C9	0.0706 (15)	0.0571 (12)	0.0775 (15)	0.0205 (11)	0.0267 (12)	−0.0019 (11)
C10	0.0529 (13)	0.0686 (15)	0.0980 (18)	0.0276 (11)	0.0311 (12)	0.0204 (13)
C11	0.0340 (11)	0.0771 (15)	0.0983 (17)	0.0073 (10)	0.0104 (10)	0.0121 (13)
C12	0.0386 (10)	0.0527 (11)	0.0761 (14)	0.0009 (9)	0.0108 (9)	−0.0003 (10)
C13	0.0396 (9)	0.0363 (9)	0.0476 (10)	0.0026 (7)	0.0098 (7)	−0.0004 (7)
C14	0.0414 (10)	0.0428 (10)	0.0616 (12)	0.0034 (8)	0.0074 (8)	0.0043 (9)
C15	0.0532 (12)	0.0505 (12)	0.0920 (17)	0.0147 (10)	0.0003 (11)	0.0070 (11)
C16	0.0852 (18)	0.0369 (12)	0.128 (2)	0.0097 (12)	−0.0110 (15)	0.0049 (12)
C17	0.0768 (16)	0.0395 (12)	0.131 (2)	−0.0106 (11)	−0.0125 (15)	0.0026 (13)
C18	0.0467 (11)	0.0440 (11)	0.0900 (16)	−0.0026 (9)	−0.0018 (10)	−0.0004 (10)
C19	0.0359 (9)	0.0374 (9)	0.0361 (9)	0.0048 (7)	0.0098 (7)	0.0050 (7)
C23	0.0366 (9)	0.0471 (10)	0.0401 (10)	−0.0076 (8)	0.0082 (8)	−0.0110 (8)
C25	0.0351 (9)	0.0478 (10)	0.0378 (9)	−0.0036 (7)	0.0048 (7)	−0.0078 (8)
C26	0.0349 (9)	0.0557 (11)	0.0470 (10)	−0.0004 (8)	0.0040 (8)	0.0011 (8)
C27	0.0364 (10)	0.0732 (13)	0.0590 (12)	−0.0031 (9)	0.0111 (9)	0.0023 (10)
C28	0.0350 (10)	0.0739 (14)	0.0664 (13)	0.0045 (10)	0.0069 (9)	−0.0135 (11)
C29	0.0501 (11)	0.0541 (12)	0.0721 (14)	0.0108 (9)	0.0023 (10)	−0.0056 (10)
C30	0.0512 (11)	0.0492 (11)	0.0570 (12)	−0.0008 (9)	0.0089 (9)	−0.0014 (9)

### Geometric parameters (Å, º)

**Table:**

O20—C19	1.226 (2)	C17—C18	1.379 (3)
O24—C23	1.233 (2)	C23—C25	1.492 (2)
N1—N2	1.364 (2)	C25—C30	1.385 (3)
N1—C5	1.361 (2)	C25—C26	1.388 (3)
N1—C7	1.431 (2)	C26—C27	1.383 (3)
N2—C3	1.334 (2)	C27—C28	1.374 (3)
N21—N22	1.394 (2)	C28—C29	1.374 (3)
N21—C19	1.350 (2)	C29—C30	1.381 (3)
N22—C23	1.352 (2)	C6—H6A	0.9600
N21—H21	0.88 (2)	C6—H6B	0.9600
N22—H22	0.90 (2)	C6—H6C	0.9600
C3—C13	1.474 (2)	C8—H8	0.9300
C3—C4	1.419 (2)	C9—H9	0.9300
C4—C19	1.482 (2)	C10—H10	0.9300
C4—C5	1.378 (2)	C11—H11	0.9300
C5—C6	1.486 (3)	C12—H12	0.9300
C7—C8	1.374 (3)	C14—H14	0.9300
C7—C12	1.378 (3)	C15—H15	0.9300
C8—C9	1.383 (3)	C16—H16	0.9300
C9—C10	1.366 (3)	C17—H17	0.9300
C10—C11	1.374 (3)	C18—H18	0.9300
C11—C12	1.386 (3)	C26—H26	0.9300
C13—C18	1.384 (3)	C27—H27	0.9300
C13—C14	1.385 (3)	C28—H28	0.9300
C14—C15	1.377 (3)	C29—H29	0.9300
C15—C16	1.362 (3)	C30—H30	0.9300
C16—C17	1.369 (4)		
			
O20···N22	2.690 (3)	C8···H6A	3.0300
O20···C13	3.413 (3)	C8···H6C	2.8800
O20···C14	3.246 (3)	C9···H12^ii^	3.0600
O20···C23	3.258 (3)	C12···H9^iv^	3.0400
O20···N21^i^	2.925 (3)	C13···H6B^ii^	3.0100
O24···N22^ii^	2.864 (3)	C14···H6B^ii^	2.9700
O24···C26^ii^	3.420 (3)	C19···H6B	2.9000
O24···C19	3.205 (3)	C19···H21^i^	2.88 (2)
O24···N21	2.656 (3)	C19···H14	2.6200
O20···H28^iii^	2.8400	C23···H26^ii^	2.9500
O20···H14	2.8000	C23···H22^ii^	2.78 (2)
O20···H6A^i^	2.8600	C25···H26^ii^	3.0100
O20···H21^i^	2.05 (2)	C26···H22	2.649 (19)
O24···H30	2.5100	C27···H15^v^	2.9100
O24···H14	2.6800	C28···H15^v^	2.9100
O24···H21	2.793 (19)	C30···H26^ii^	3.0200
O24···H22^ii^	1.98 (2)	H6A···C8	3.0300
O24···H26^ii^	2.6600	H6A···H8	2.4200
N2···C8^i^	3.428 (3)	H6A···H16^vi^	2.3800
N21···O24	2.656 (3)	H6A···O20^ii^	2.8600
N21···C6	3.257 (3)	H6B···N21	2.8100
N21···O20^ii^	2.925 (3)	H6B···C19	2.9000
N22···O20	2.690 (3)	H6B···C3^i^	3.0600
N22···O24^i^	2.864 (3)	H6B···C13^i^	3.0100
N2···H12	2.7300	H6B···C14^i^	2.9700
N2···H10^iv^	2.7600	H6C···C7	2.8300
N2···H18	2.6800	H6C···C8	2.8800
N21···H6B	2.8100	H8···C5	2.9400
N22···H26	2.6200	H8···C6	2.8100
N22···H29^v^	2.8200	H8···H6A	2.4200
C3···C6^ii^	3.549 (3)	H8···C3^ii^	2.9000
C3···C8^i^	3.594 (4)	H9···C12^vii^	3.0400
C6···C13^i^	3.584 (3)	H10···N2^vii^	2.7600
C6···C3^i^	3.549 (3)	H10···H18^vii^	2.5900
C6···C8	3.167 (3)	H12···N2	2.7300
C6···N21	3.257 (3)	H12···C9^i^	3.0600
C8···C3^ii^	3.594 (4)	H14···O20	2.8000
C8···N2^ii^	3.428 (3)	H14···O24	2.6800
C8···C6	3.167 (3)	H14···C4	2.8400
C13···O20	3.413 (3)	H14···C19	2.6200
C13···C6^ii^	3.584 (3)	H15···C27^viii^	2.9100
C14···C19	3.193 (3)	H15···C28^viii^	2.9100
C14···O20	3.246 (3)	H16···H6A^ix^	2.3800
C19···C14	3.193 (3)	H18···N2	2.6800
C19···O24	3.205 (3)	H18···H10^iv^	2.5900
C23···O20	3.258 (3)	H21···O24	2.793 (19)
C23···C26^ii^	3.529 (3)	H21···C5	2.923 (19)
C25···C29^iii^	3.439 (4)	H21···C6	3.04 (2)
C25···C26^ii^	3.494 (3)	H21···O20^ii^	2.05 (2)
C26···C25^i^	3.494 (3)	H21···C19^ii^	2.88 (2)
C26···C23^i^	3.529 (3)	H22···C26	2.649 (19)
C26···O24^i^	3.420 (3)	H22···H26	2.1500
C29···C25^iii^	3.439 (4)	H22···O24^i^	1.98 (2)
C29···C30^iii^	3.510 (4)	H22···C23^i^	2.78 (2)
C30···C29^iii^	3.510 (4)	H26···N22	2.6200
C30···C30^iii^	3.514 (3)	H26···H22	2.1500
C3···H6B^ii^	3.0600	H26···O24^i^	2.6600
C3···H8^i^	2.9000	H26···C23^i^	2.9500
C4···H14	2.8400	H26···C25^i^	3.0100
C5···H8	2.9400	H26···C30^i^	3.0200
C5···H21	2.923 (19)	H28···O20^iii^	2.8400
C6···H21	3.04 (2)	H29···N22^viii^	2.8200
C6···H8	2.8100	H30···O24	2.5100
C7···H6C	2.8300		
			
N2—N1—C5	112.61 (12)	C26—C25—C30	118.78 (15)
N2—N1—C7	118.98 (12)	C25—C26—C27	120.52 (17)
C5—N1—C7	128.39 (13)	C26—C27—C28	120.01 (18)
N1—N2—C3	104.87 (12)	C27—C28—C29	119.97 (18)
N22—N21—C19	118.85 (15)	C28—C29—C30	120.31 (19)
N21—N22—C23	118.28 (15)	C25—C30—C29	120.41 (17)
N22—N21—H21	115.4 (12)	C5—C6—H6A	109.00
C19—N21—H21	121.6 (12)	C5—C6—H6B	109.00
N21—N22—H22	115.1 (12)	C5—C6—H6C	109.00
C23—N22—H22	125.1 (12)	H6A—C6—H6B	110.00
N2—C3—C4	110.78 (14)	H6A—C6—H6C	109.00
N2—C3—C13	119.82 (14)	H6B—C6—H6C	110.00
C4—C3—C13	129.13 (14)	C7—C8—H8	120.00
C3—C4—C5	105.69 (14)	C9—C8—H8	120.00
C5—C4—C19	127.26 (14)	C8—C9—H9	120.00
C3—C4—C19	126.92 (14)	C10—C9—H9	120.00
N1—C5—C4	106.04 (13)	C9—C10—H10	120.00
N1—C5—C6	123.90 (14)	C11—C10—H10	120.00
C4—C5—C6	130.04 (15)	C10—C11—H11	120.00
C8—C7—C12	120.55 (16)	C12—C11—H11	120.00
N1—C7—C12	119.23 (15)	C7—C12—H12	121.00
N1—C7—C8	120.21 (14)	C11—C12—H12	121.00
C7—C8—C9	119.73 (17)	C13—C14—H14	120.00
C8—C9—C10	120.4 (2)	C15—C14—H14	120.00
C9—C10—C11	119.6 (2)	C14—C15—H15	120.00
C10—C11—C12	120.9 (2)	C16—C15—H15	120.00
C7—C12—C11	118.80 (19)	C15—C16—H16	120.00
C3—C13—C14	120.41 (15)	C17—C16—H16	120.00
C3—C13—C18	120.93 (15)	C16—C17—H17	120.00
C14—C13—C18	118.53 (16)	C18—C17—H17	120.00
C13—C14—C15	120.41 (17)	C13—C18—H18	120.00
C14—C15—C16	120.4 (2)	C17—C18—H18	120.00
C15—C16—C17	120.1 (2)	C25—C26—H26	120.00
C16—C17—C18	120.2 (2)	C27—C26—H26	120.00
C13—C18—C17	120.43 (19)	C26—C27—H27	120.00
N21—C19—C4	114.28 (14)	C28—C27—H27	120.00
O20—C19—N21	122.18 (15)	C27—C28—H28	120.00
O20—C19—C4	123.53 (14)	C29—C28—H28	120.00
O24—C23—C25	121.98 (15)	C28—C29—H29	120.00
N22—C23—C25	117.13 (14)	C30—C29—H29	120.00
O24—C23—N22	120.88 (15)	C25—C30—H30	120.00
C23—C25—C26	123.81 (15)	C29—C30—H30	120.00
C23—C25—C30	117.41 (15)		
			
C5—N1—N2—C3	0.71 (18)	C5—C4—C19—O20	125.89 (19)
C7—N1—N2—C3	−177.81 (14)	C5—C4—C19—N21	−53.0 (2)
N2—N1—C5—C4	−0.77 (18)	N1—C7—C8—C9	−179.50 (17)
N2—N1—C5—C6	−179.13 (14)	C12—C7—C8—C9	1.4 (3)
C7—N1—C5—C4	177.58 (15)	N1—C7—C12—C11	−179.31 (18)
C7—N1—C5—C6	−0.8 (3)	C8—C7—C12—C11	−0.2 (3)
N2—N1—C7—C8	−132.39 (16)	C7—C8—C9—C10	−1.4 (3)
N2—N1—C7—C12	46.7 (2)	C8—C9—C10—C11	0.1 (3)
C5—N1—C7—C8	49.4 (2)	C9—C10—C11—C12	1.1 (4)
C5—N1—C7—C12	−131.54 (19)	C10—C11—C12—C7	−1.1 (3)
N1—N2—C3—C4	−0.36 (18)	C3—C13—C14—C15	−175.98 (17)
N1—N2—C3—C13	−174.96 (14)	C18—C13—C14—C15	−0.2 (3)
C19—N21—N22—C23	81.5 (2)	C3—C13—C18—C17	175.0 (2)
N22—N21—C19—O20	10.8 (2)	C14—C13—C18—C17	−0.8 (3)
N22—N21—C19—C4	−170.27 (14)	C13—C14—C15—C16	1.0 (3)
N21—N22—C23—O24	7.9 (2)	C14—C15—C16—C17	−0.8 (4)
N21—N22—C23—C25	−173.20 (15)	C15—C16—C17—C18	−0.1 (5)
N2—C3—C4—C5	−0.09 (19)	C16—C17—C18—C13	0.9 (4)
N2—C3—C4—C19	176.06 (15)	O24—C23—C25—C26	164.22 (17)
C13—C3—C4—C5	173.87 (16)	O24—C23—C25—C30	−16.3 (2)
C13—C3—C4—C19	−10.0 (3)	N22—C23—C25—C26	−14.7 (2)
N2—C3—C13—C14	145.43 (16)	N22—C23—C25—C30	164.83 (16)
N2—C3—C13—C18	−30.3 (3)	C23—C25—C26—C27	178.06 (16)
C4—C3—C13—C14	−28.1 (3)	C30—C25—C26—C27	−1.4 (3)
C4—C3—C13—C18	156.25 (19)	C23—C25—C30—C29	−178.60 (17)
C3—C4—C5—N1	0.50 (17)	C26—C25—C30—C29	0.9 (3)
C3—C4—C5—C6	178.72 (16)	C25—C26—C27—C28	0.8 (3)
C19—C4—C5—N1	−175.63 (15)	C26—C27—C28—C29	0.4 (3)
C19—C4—C5—C6	2.6 (3)	C27—C28—C29—C30	−0.9 (3)
C3—C4—C19—O20	−49.4 (2)	C28—C29—C30—C25	0.2 (3)
C3—C4—C19—N21	131.63 (17)		

Symmetry codes: (i) *x*, −*y*+1/2, *z*+1/2; (ii) *x*, −*y*+1/2, *z*−1/2; (iii) −*x*, −*y*+1, −*z*+1; (iv) −*x*+1, *y*+1/2, −*z*+1/2; (v) −*x*, *y*−1/2, −*z*+1/2; (vi) *x*, *y*−1, *z*; (vii) −*x*+1, *y*−1/2, −*z*+1/2; (viii) −*x*, *y*+1/2, −*z*+1/2; (ix) *x*, *y*+1, *z*.

### Hydrogen-bond geometry (Å, º)

**Table:**

*D*—H···*A*	*D*—H	H···*A*	*D*···*A*	*D*—H···*A*
N21—H21···O20^ii^	0.88 (2)	2.05 (2)	2.925 (3)	174.5 (17)
N22—H22···O24^i^	0.90 (2)	1.98 (2)	2.864 (3)	167.7 (17)

Symmetry codes: (i) *x*, −*y*+1/2, *z*+1/2; (ii) *x*, −*y*+1/2, *z*−1/2.
